# Classification of VLF/LF Lightning Signals Using Sensors and Deep Learning Methods

**DOI:** 10.3390/s20041030

**Published:** 2020-02-14

**Authors:** Jiaquan Wang, Qijun Huang, Qiming Ma, Sheng Chang, Jin He, Hao Wang, Xiao Zhou, Fang Xiao, Chao Gao

**Affiliations:** 1 School of Physics and Technology, Wuhan University, Wuhan 430072, China; 2 Institute of Electrical Engineering, Chinese Academy of Sciences, Beijing 100190, China; 3 University of Chinese Academy of Sciences, Beijing 100049, China

**Keywords:** VLF/LF lightning waveform, automatic classification, deep learning, convolutional neural network (CNN)

## Abstract

Lightning waveform plays an important role in lightning observation, location, and lightning disaster investigation. Based on a large amount of lightning waveform data provided by existing real-time very low frequency/low frequency (VLF/LF) lightning waveform acquisition equipment, an automatic and accurate lightning waveform classification method becomes extremely important. With the widespread application of deep learning in image and speech recognition, it becomes possible to use deep learning to classify lightning waveforms. In this study, 50,000 lightning waveform samples were collected. The data was divided into the following categories: positive cloud ground flash, negative cloud ground flash, cloud ground flash with ionosphere reflection signal, positive narrow bipolar event, negative narrow bipolar event, positive pre-breakdown process, negative pre-breakdown process, continuous multi-pulse cloud flash, bipolar pulse, skywave. A multi-layer one-dimensional convolutional neural network (1D-CNN) was designed to automatically extract VLF/LF lightning waveform features and distinguish lightning waveforms. The model achieved an overall accuracy of 99.11% in the lightning dataset and overall accuracy of 97.55% in a thunderstorm process. Considering its excellent performance, this model could be used in lightning sensors to assist in lightning monitoring and positioning.

## 1. Introduction

Lightning is one of the most commonly geophysical phenomena [1]. There are 30–100 lightning strikes per second worldwide. In 2013, the World Wide Lightning Location Network (WWLLN) detected 210.6 million lightning strikes through 67 sites [2]. Lightning is simply divided into two types according to the location where the lightning occurs: cloud flash and cloud-to-ground flash (CG).

**Figure 1:**
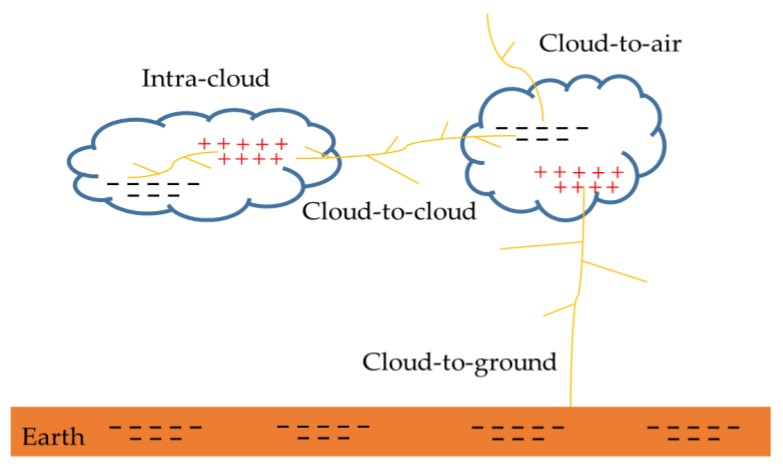
Different types of the lightning discharge.

As shown in Figure 1, cloud flash includes cloud-to-cloud discharges, intra-cloud discharges, and cloud-to-air discharges. According to statistics, cloud flashes account for about 75% of global lightning events [3]. When these discharges occur from cloud to ground, it is called cloud-to-ground discharges. This discharge is the most harmful to human production and life, and can directly lead to human death. In general, about 90% of cloud-to-ground flashes are negative cloud-to-ground flashes (−CG), and the remaining 10% are positive cloud-to-ground flashes (+CG) [4,5]. Narrow bipolar events (NBE) is usually generated during a thunderstorm [6,7,8]. The discharge channel is short, and the intensity is stronger than the cloud-to-ground flash or normal cloud flash. Lightning’s seemingly random occurrence in space and time and the wide range of its significant time variation makes lightning particularly difficult to study. Different discharge processes produce different electromagnetic radiation. The accurate classification of lightning is of great significance for lightning observation, lightning disaster investigation, and research on the rules of lightning activity.

The lightning process is accompanied by strong electromagnetic radiation, and the signal frequency bands cover very low frequency (VLF), low frequency (LF), high frequency (HF), and very high frequency (VHF). The main method for observing the lightning process is to use antennas to receive lightning signals in different frequency bands [9,10,11,12]. Since the 1980s, many regional lightning monitoring networks have been deployed around the world. Distinguishing cloud flashes from cloud ground flashes has been an important indicator of lightning monitoring and research. According to incomplete statistics, more than 10,000 people are killed or injured each year due to lightning strikes, and cloud-to-ground flashes are the direct cause of lightning strikes. After receiving the lightning signal, the lightning sensor will classify the signal, determine whether it is a cloud flash or cloud ground flash, and upload the classification result to the lightning location calculation server. Generally, the rise and fall time of the lightning waveform and the signal-to-noise ratio of the waveform are calculated to distinguish between cloud flash and cloud ground flash [13,14]. Due to the complexity and diversity of lightning occurrences, the statistical method is only suitable for single-pulse events or a specific thunderstorm. In addition, the classification effect of this method is not ideal in practical applications: the recognition rate of cloud ground flashes is 70% ~ 80%, and the recognition rate of cloud flashes is less than 85%, and it cannot effectively distinguish the ionosphere reflection signals [15].

From the perspective of lightning location, cloud-to-ground flash is the lightning that hits the ground directly. If the location algorithm can use the type of lightning as a known condition, this will have an important effect on improving the location accuracy. Lightning signal classification plays an important role in a lightning warning and weather forecasting. A large number of cloud-to-ground flashes herald regional thunderstorms. Accurate identification of cloud-to-light types can improve aircraft navigation safety. In summary, we need a better way to distinguish lightning signals.

Deep learning [16] is a rapidly growing area of machine learning, which has been widely used in the fields of image recognition, speech analysis, and medical diagnosis in recent years [17,18,19,20]. Recently, Zaiwar Ali et al. [21] proposed an energy-saving deep learning shunting scheme to train a deep learning-based intelligent decision-making algorithm; Mohamed Alloghani et al. [22] applied machine learning algorithms to the clustering and prediction of vital signs; Stamatios Samaras et al. [23] described the progress of deep learning of multi-sensor information fusion in drone applications. Heena Rathore et al. [24] proposed a new deep learning strategy for classifying different attack methods of deep brain implants. The proposed method was able to effectively detect different types of simulated attack patterns, thereby notifying patients of possible attacks. The effect of deep brain stimulation was tested in patients with Parkinson’s tremor. Dengshan Li et al. [25] proposed a method for detecting rice diseases and insect pests based on deep convolutional neural networks. Ammar Mahmood et al. [26] used the deep residual function to automatically classify kelp. These works strongly demonstrated the powerful role of deep learning algorithms in various research fields. Considering that the lightning signal and the ECG signal have a high characteristic similarity, they are both pulse signals, and deep learning has been applied to the ECG signal processing. Therefore, the use of deep learning methods to classify lightning is of great significance for analyzing the lightning process and investigating lightning disasters.

### Novelty and Contributions

In this study, we proposed a novel method for classifying lightning signals based on a one-dimensional convolutional deep neural network. To our knowledge, this was the first attempt in the classification of lightning signals. One-dimensional convolutional neural network (1D-CNN) is able to identify patterns and learn useful information from raw input data. 1D-CNN does not require extensive data preprocessing, making it particularly suitable for analyzing lightning data. In addition, since the performance of 1D-CNN tends to improve with the increase in the amount of training data, this method can be well applied to a large number of lightning data classifications. We analyzed a large amount of lightning waveform data, and the related work is summarized as follows:

- Using the lightning waveforms and lightning location data from different regions collected by the three-dimensional lightning positioning network of the Institute of Electrical Engineering of the Chinese Academy of Sciences, a lightning waveform database was constructed for deep learning testing, training, and verification. The collected lightning signal is an electric field signal in the VLF/LF band.
- Based on the expert classification method, we classified the waveforms in the lightning waveform database. Currently, the database contains 10 types of lightning. It needs to be emphasized that we considered lightning signals transmitted from different distances, including but not limited to the basic types of lightning described earlier. This paper mainly analyzed the feasibility and effectiveness of deep learning methods in lightning signal classification.
- A lightning classification algorithm based on deep learning was proposed to replace the statistical method. The input of the algorithm is a fixed-length lightning signal, and the output is a lightning type number.
- Apply the classification algorithm to a thunderstorm process to detect the actual application of the algorithm. Test results showed that the model could accurately recognize lightning during this thunderstorm as high as 97.55%.

The paper is organized as follows. Section 2 introduces the lightning dataset and data preprocessing method. Section 3 introduces some of the deep learning methods used for classification. Section 4 details the model structure and parameters, model evaluation, and model test results. Section 5 compares the convolutional neural network with a statistical method. It also discusses the impact of different model structures on classification performance and analyzes the real-time processing performance of the model. The conclusions and future research directions are given in Section 6.

## 2. Dataset and Preprocessing

### 2.1. Lightning Dataset

**Figure 2:**
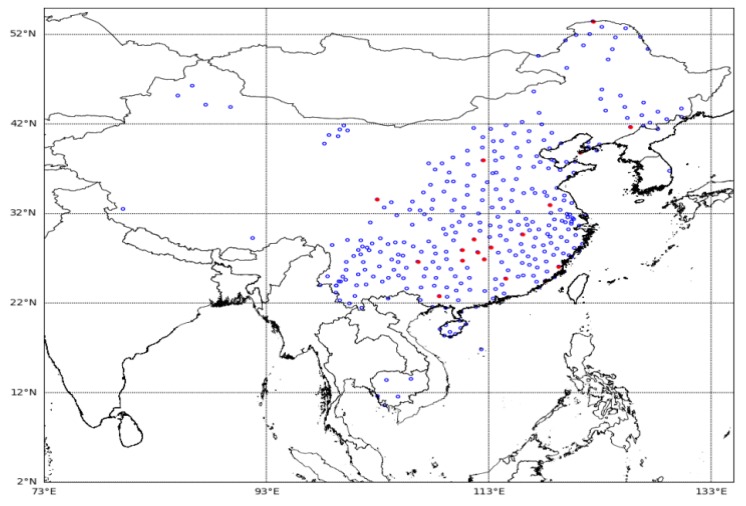
Map of lightning detection sites. The blue circle represents a regular site, and the red circle represents a waveform acquisition site.

Lightning data comes from the three-dimensional lightning location system (advanced direction-time lightning detection system, ADTD) of the Institute of Electrical Engineering, Chinese Academy of Sciences. The system has 333 detection stations, and the detection region covers China and Southeast Asia. The site distribution is shown in Figure 2. The positioning error is less than 500 m. Each detection site includes a VLF/LF electric field antenna, a magnetic antenna, a GPS receiving system, and a signal processing system. The channel bandwidth of the device is 3 kHz to 400 kHz, and the GPS time synchronization accuracy is less than 20 ns.

Since 2018, we have upgraded some ADTD lightning detectors. The new detector has the functions of lightning waveform acquisition, storage, and remote file transfer. The system adopts the trigger sampling method, the sampling rate is 1MSPS (Million Samples per Second), the sampling time length is 1 ms, and the pre-trigger length is 100 μs.

**Figure 3:**
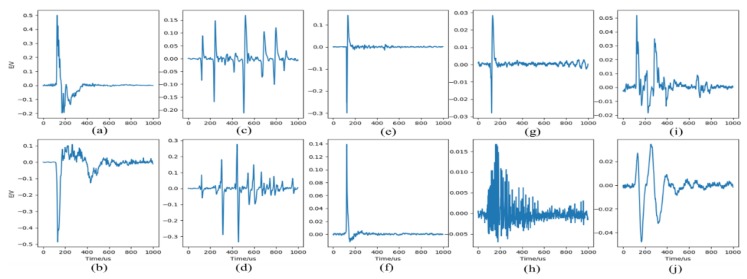
Example of some typical very low frequency/low frequency (VLF/LF) electric field waveforms. (**a**) negative cloud-to-ground flash (−CG), (**b**) positive CG (+CG), (**c**) negative pre-breakdown (−PBP), (**d**) positive PBP (+PBP), (**e**) negative narrow bipolar event (−NBE), (**f**) positive NBE (+NBE), (**g**) NBE, (**h**) multi-pulse cloud flash (MP), (**i**) cloud ground flash with ionosphere reflected signals (CG-IR), (**j**) skywave (SW).

The lightning signals received by the lightning detector divide into the following categories: cloud ground flashes and cloud flashes, where cloud ground flashes include: positive cloud-to-ground flash (+CG), negative cloud-to-ground flash (−CG), cloud ground flash with ionosphere reflected signals (CG-IR); cloud flash includes positive NBE (+NBE), negative NBE (−NBE), positive pre-breakdown (+PBP), negative pre-breakdown (−PBP), multi-pulse cloud flash (MP), polarity pulse (NBE, positive and negative amplitudes are consistent), far-field skywave (SW). The typical waveforms of these types are shown in Figure 3. The total number of signal samples is 50,000, and the number of samples for each type is 5000. Please refer to the Supplementary Materials for detailed data.

### 2.2. Pre-Processing

**Figure 4:**
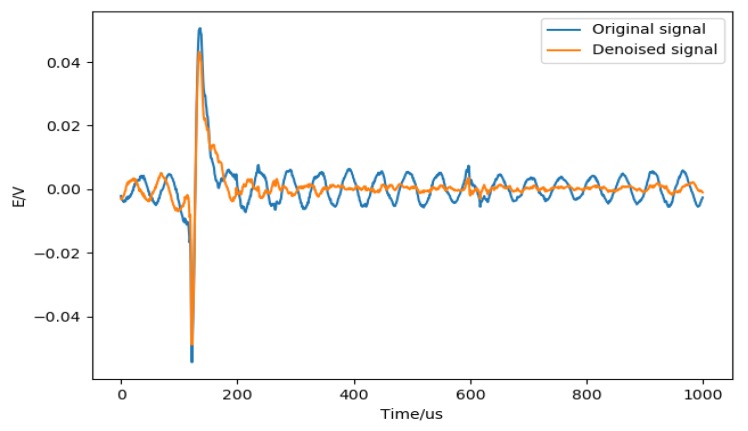
An example of zero-phase digital filtering. The blue line indicates data before filtering, and the orange line indicates data after filtering.

Data preprocessing is mainly divided into two parts:

(1) Digital filter to reduce noise. The zero-phase digital filtering [27] method is used to remove the high-frequency noise contained in the signal. Zero-phase filtering reduces noise in the signal and preserves the lightning complex at the same time it occurs in the original. Conventional filtering reduces noise in the signal but delays the lightning complex. Figure 4 shows an example of suppressing signal noise by zero-phase digital filtering.
(2) Data standardization (normalization) [28]. Data standardization is to scale the data to a specific interval according to a certain algorithm, remove the unit limit of the data, and convert it into a dimensionless pure value. Normally, standardization allows the features between different dimensions to be numerically comparable, which can greatly improve the accuracy of the classifier and prompt the convergence speed. The most typical one is the normalization of the data; that is, the data is uniformly mapped to [0,1]. Data normalization methods include min-max normalization (min-max normalization), log conversion, arctangent conversion, z-score normalization, and fuzzy quantization.

In this study, the z-score normalization method was used. For a sample *x* of length *n*, the calculation formula is:

(1) $$x^{\prime }=\frac{x-\mu }{\sigma }$$

(2) $$\mu =\frac{1}{n}\overset{n}{\underset{i=1}{\sum }}a_{i}$$

(3) $$\sigma =\sqrt{\frac{1}{n}\overset{n}{\underset{i=1}{\sum }}{\left(a_{i}-\mu \right)}^{2}}$$

where *μ* is the average of sample data, *σ* is the standard deviation of sample data, and $a_{i}$ is the amplitude of the *i*th point.

## 3. Methods

**Figure 5:**
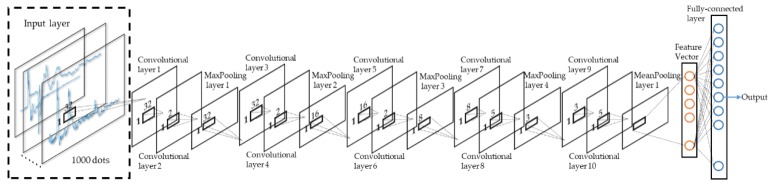
Proposed one-dimensional convolutional neural network (1D-CNN) structure diagram composed of an input layer, ten convolutional layers, five pooling layers, one fully-connected layer, and output.

Lightning signals are similar to signals, such as voice and ECG, and belong to one-dimensional time series. This paper built a one-dimensional CNN model (as shown in Figure 5). The input data of the model was waveform data after filtering and noise reduction and standard deviation normalization, and each data consisted of 1000 sampling points. The sampling rate of the sensor was 1MSPS, and each point represented a time length of 1 μs, that is, 1000 points represented that the sensor collected information with a length of 1 ms. The model consisted of multiple one-dimensional convolutional layers and pooling layers (maximum pooling and mean pooling). The specific parameters of the model are detailed in Section 4.1.

### 3.1. Independent Feature Branch

With the rapid development of deep learning methods, many advanced algorithms have been applied to deep learning methods. Researchers apply different algorithms and network structures to different fields. Lightning signals are instantaneous signals. For signal classification detection, it is mainly to identify the relevant characteristics of the signal at a certain moment. Therefore, the feature extraction of the signal is crucial. This section briefly introduces several deep learning methods used in the 1D-CNN lightning signal classification model. Among them, one-dimensional convolution was used to extract the characteristics of the lightning signal; the pooling method down-sampled the signal to improve the calculation speed; the fully connected layer integrated all features, and finally gave the probability distribution of the signal on different types. Each layer was from top to bottom to complete the classification of lightning signals.

#### 3.1.1. 1-D Convolution Layer

The 1-D convolution layer performs 1-D convolution, data filling, and activation calculations. Similar to using 2-D convolution to extract image features, 1-D convolution calculations are used to extract lightning waveform features. Through the calculation of convolution kernels of different sizes, the lightning waveform characteristics of different scales are extracted. The activation function can add some non-linear factors to the network, causing the network to better solve multiple complex problems. The operation can be formulated as:

(4) $$X_{j}^{l}=f\left(X_{i}^{l-1}*K_{ij}^{l}+b_{j}^{l}\right)$$

where $K_{ij}^{l}$ is a 1-D kernel (or weight) connecting $X_{j}^{l}$ and $X_{i}^{l-1}$, * represents a 1-D convolution operation, $b_{j}^{l}$ is the corresponding deviation, and *f* (⋯) is the activation function, producing nonlinearity; here, a nonlinearity rectification function (rectified linear unit, ReLU) [16] is used, *f* (*x*) = max (0, *x*). It is known as an efficient activation function for the deep learning structure, which can accelerate the training process [29].

#### 3.1.2. 1-D Pooling Layer

The pooling layer (also called down-sampling) can reduce the dimensionality of the feature extracted by the convolutional layer. On the one hand, the feature map is smaller, simplifying the computational complexity of the network [30] and avoiding over-fitting to a certain extent; on the other hand, feature compression is performed to extract the main features.

Considering the transient nature of the lightning signal and the inconsistency of the time scale, the pooling layer can effectively reduce the influence of these factors, greatly reduce the amount of data calculation, and improve the robustness of the model. Common pooling methods include maximum pooling and mean pooling. In the 1-D CNN lightning signal classification model, the maximum pooling and mean pooling are used in combination to obtain signal characteristics at different scales.

#### 3.1.3. Fully-Connected Layer

Fully-connected layer plays the role of “classifier” in the entire convolutional neural network. The fully connected layer combines the features of different scales of the lightning waveform extracted by the convolutional layer and the pooling layer and makes a decision on all the extracted features from a global perspective. The calculation can be expressed as:

(5) Y=f(X⋅W+b)

where *W* is a weight matrix created by the network layer, b is a bias vector, and *f* (⋯) is an activation function. Here, the softmax function is used to calculate the prediction probability of each category.

### 3.2. Parameter Optimization

Deep learning has derived various parameter optimization methods, which can trace back to the stochastic gradient descent (SGD) algorithm [16]. In this paper, Adam [31] (adaptive momentum) adaptive momentum was used to replace the SGD algorithm. Adam combines the best performance of the AdaGrad [32] and RMSProp, using both first-order and second-order momentum. 

Adam’s tuning is relatively simple, and the default parameters can handle most problems. There are only two hyperparameters: $\beta_{1}$ and $\beta_{2}$, the former controls first-order momentum, and the latter controls second-order momentum. Adam optimizer parameters default to $\beta_{1}=0.9$ and $\beta_{2}=0.999$, the initial learning rate is 0.001, and the size of each training batch is 100.

## 4. Result

### 4.1. Model Structure and Parameters

Based on the above hierarchical structure and training methods, parameters were determined through experiments, such as the size of the convolution kernel and the number of convolution layers. Finally, the model structure and parameters were determined, as shown in Table 1. Due to the wide spectrum of lightning signals, the signal characteristics appeared as pulse widths of different scales in the time domain. Therefore, when designing the size of the convolution kernel, the kernel size of the convolution layer decreased with the number of layers, and the recognition scale changed from large when it was small, and it finally achieved fine identification of the signal. The model and experiments were implemented in Keras + TensorFlow on a Windows PC with Intel Core i7 CPU (@ 2.2 GHz) and NVIDIA Quadro P600.

### 4.2. Train and Evaluate

A certain number of samples were randomly drawn from the entire data set for training, and the remaining part was used for testing. In this paper, 5-fold cross-validation was used for model tuning to find hyperparameter values that optimize the generalization performance of the model. Lightning data set was divided into five equal sets, and each set was used once as testing data, and the remaining sets were used as training data. As for multiple types of classification models, the accuracy of each category was used to evaluate model performance:

(6) $$\mathrm{Accuracy}=\frac{CorrectPredictions}{TotalSamples}$$

**Figure 6:**
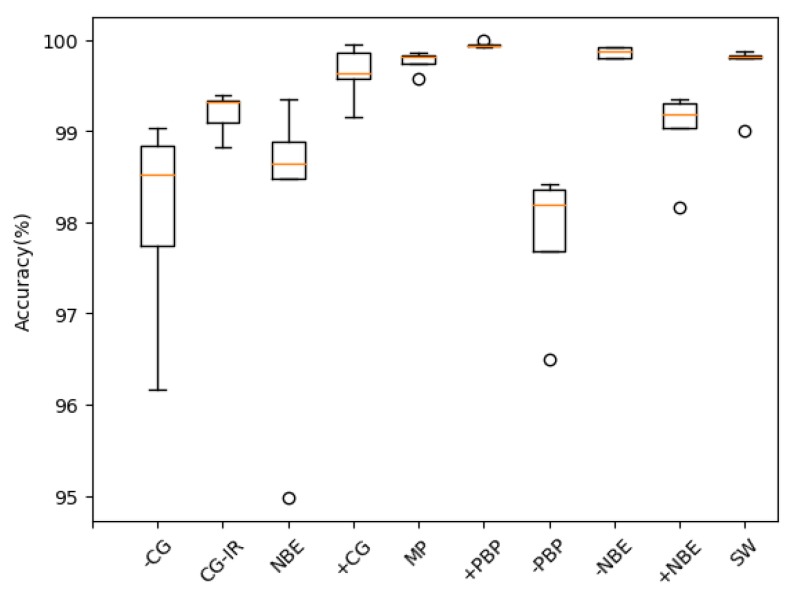
Box plot of model classification accuracy.

**Figure 7:**
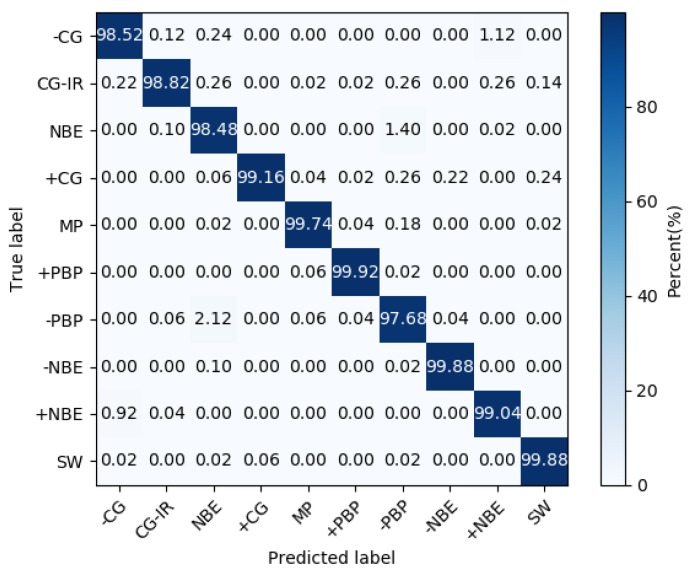
Confusion matrix for classification results.

Table 2 lists the model’s recognition accuracy for different types of lightning under each folding cross-validation (K). Figure 6 shows the box plot of model classification accuracy. Figure 7 shows the model’s confusion matrix for different types of recognition results during five-fold cross-validation.

It could be seen from Table 2 that for the 10 types of lightning signals, the model had good recognition, and the overall recognition accuracy rate was as high as 99.11%. From the confusion matrix, it could be seen that for several types of signals that were prone to confusion (such as −CG and +NBE, NBE, and PBP), the model still had good discrimination. The average recognition accuracy of a single type was as low as 97.83%, and the highest was 99.95%.

### 4.3. Model Test

**Figure 8:**
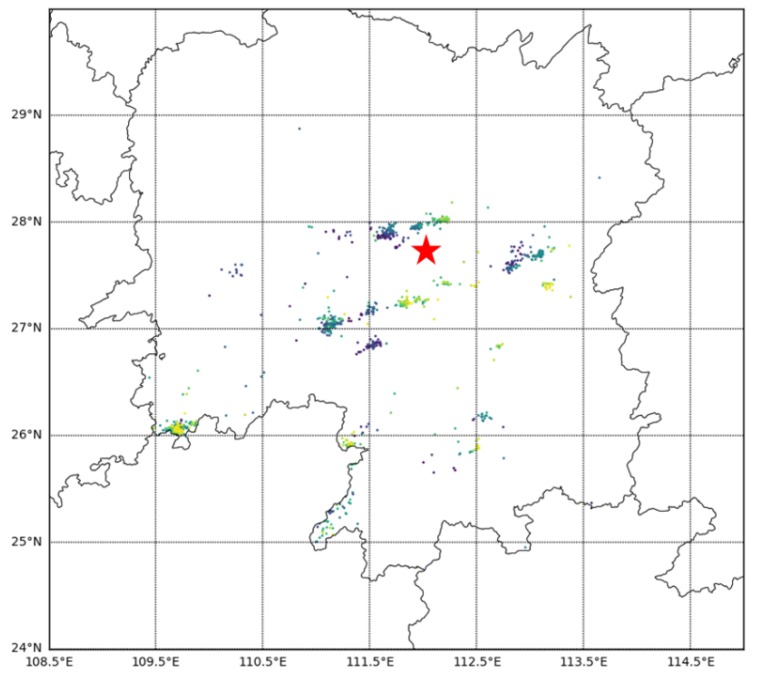
Thunderstorm map. The red pentagram represents a site location.

Several thunderstorms occurred in Hunan, China, on 6 June 2019. Figure 8 shows the distribution of thunder and lightning at 13:00 on that day. The lightning waveform data was recorded by the lightning detection station located in Loudi, Hunan Province. A total of 9647 pieces of data were collected by the detection station within an hour and used for testing.

The model classification results are shown in Table 3. Through manual inspection, the model’s recognition accuracy of various signals was given. The overall classification accuracy of the model was 97.55%. For most categories, the classification accuracy was above 97%.

Besides, it could be noted that the accuracy of +CG was significantly lower than in other classes. There were two factors:

(1) The +CG occurrence probability was significantly less than other classes, which resulted in fewer samples in this class.
(2) We found that there were a few data that were significantly different from the waveforms in the data set when manually verifying the classification accuracy of the model. This was also the reason that the accuracy of +CG type recognition was significantly reduced.

Overall, the model had a good classification effect on these 10 types of lightning waveforms.

## 5. Discussion

### 5.1. Methods Comparison

This section has discussed the technical advantages of using convolutional neural networks over traditional statistical methods in the field of lightning signal classification in detail. The comparison method refers to the typical characteristics of the three types of lightning signals counted by Li Cai [33]. Three types of lightning signals include cloud-to-ground flash, cloud-to-cloud flash, and +NBE/−NBE. In order to distinguish these types of lightning, specific nine waveform parameter extraction methods are used to classify the collected lightning radiation electric field waveforms.

**Figure 9:**
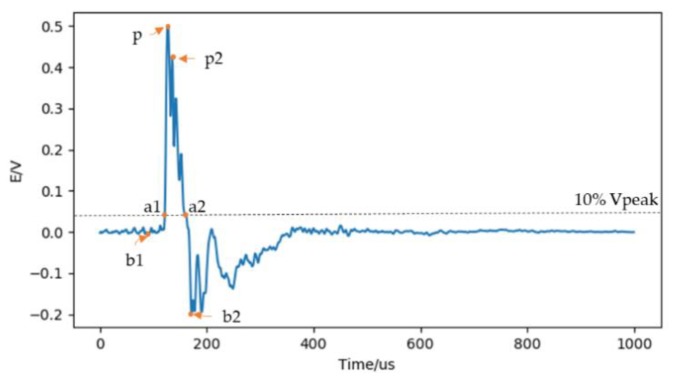
Schematic diagram of waveform characteristic parameter definition.

As shown in Figure 9, they are:

(1) Pulse rise time. The time elapsed from the 10% peak of the waveform to the peak of the waveform. $T_{r}=T_{P}-T_{a1}$.
(2) Pulse fall time. The time elapsed from the peak of the waveform to the 10% peak of the waveform. $T_{f}=T_{a1}-T_{P}$.
(3) Pulse width. The time elapsed from the 10% peak of the rising edge to the 10% peak of the falling edge. $T_{PW}=T_{a2}-T_{a1}=T_{r}+T_{f}$.
(4) Forward peak-to-peak ratio. The ratio of initial negative peak to maximum peak. $R_{1}=v_{b1}/v_{P}$.
(5) Backward peak-to-peak ratio. The ratio of following negative peak to maximum peak. $R_{2}=v_{b2}/v_{P}$.
(6) Sub-peak ratio. The ratio of the maximum peak to the secondary peak. $R_{3}=v_{p2}/v_{P}$.
(7) Signal to noise ratio (SNR). The ratio of the average power of the signal within 20 μs before and after the peak point to the average power of the signal at other times.
  $$\mathrm{SNR}=\frac{\sum_{N=P-10}^{P+10}A_{N}{}^{2}}{\sum_{N=0}^{P-10}A_{N}{}^{2}+\sum_{N=P+10}^{1000}A_{N}{}^{2}}$$
  where $A_{N}$ represents the amplitude of the Nth point, P represents the point with the largest amplitude.
(8) Pre-SNR. The ratio of the average power of the signal within 20 μs before and after the peak point to the average power of the signal before the peak point.
  $$\mathrm{preSNR}=\frac{\sum_{N=P-10}^{P+10}A_{N}{}^{2}}{\sum_{N=0}^{P-10}A_{N}{}^{2}}$$
(9) Post-SNR. The ratio of the average power of the signal within 20 μs before and after the peak point to the average power of the signal after the peak point.
  $$\mathrm{postSNR}=\frac{\sum_{N=P-10}^{P+10}A_{N}{}^{2}}{\sum_{N=P+10}^{1000}A_{N}{}^{2}}$$

We strictly calculated the 50,000 lightning waveform data that have been classified in the database, according to Li Cai’s statistical law. The related calculation results are shown in Table 4.

We newly added a classification called Other Types, which represents signals whose calculation results are not any of the three types (cloud-to-cloud flash, cloud-to-ground flash, and +NBE/−NBE). This article separates unipolar narrow pulse events separately into +NBE and −NBE. It should be emphasized that there is a category in this paper called a narrow bipolar pulse event, which is very different from Li Cai’s. The distinguishing feature is that the positive and negative amplitudes of these signals are symmetrical.

The results showed that compared to the accuracy of classifying waveforms (99.11%) in the waveform library using a one-dimensional convolutional neural network (Table 2), the average classification accuracy using statistical methods was only 55.66%. In addition, there are nearly 30% of the waveform that could not be identified by statistical methods. Firstly, the statistical method is a statistical rule for a specific waveform, it has a certain dependence on the sample, and there are some differences in the statistics of different samples. Secondly, there are other types of waveforms in the waveform library, such as CG-IR, MP. The classification accuracy of these two types is particularly low, which indicates that existing statistical rules do not consider these types of signals, and then, due to the similarity between the narrow bipolar event and the cloud-based flash memory, both of them have a stronger amplitude. The method distinguishes these two types of signals with certain defects. Finally, comparing the two methods, it shows that the one-dimensional convolutional neural network has a strong advantage in the feature extraction of lightning signals. It does not need to manually extract waveform features and avoid the impact of different feature parameters on classification.

### 5.2. Model Structure Analysis

**Figure 10:**
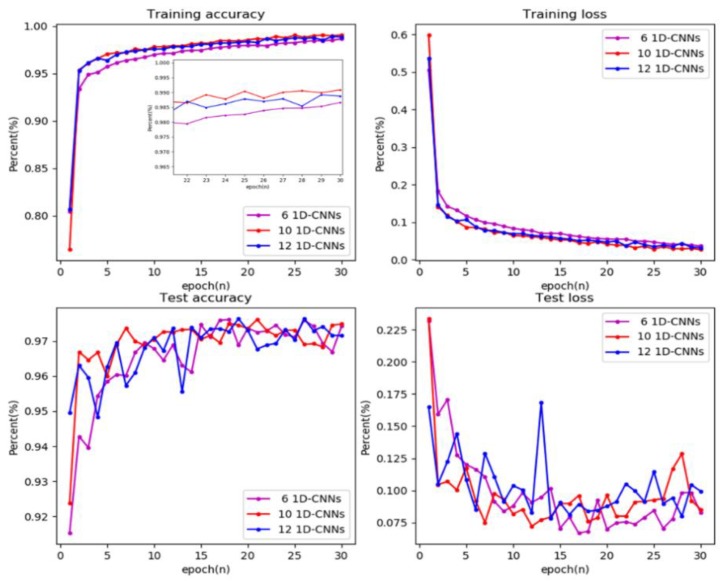
Training and test results of models with different convolutional layers.

Different model structures would lead to different experimental results. The model structure proposed in this article was a scheme selected after a large number of experiments. Because the selection of different parameters leads to too many model combinations, we selected some experimental data to illustrate the parameter selection method of the lightning classification model proposed in this paper. Figure 10 shows the results of a model with different convolutional layers under training and testing conditions. Using the 10-layer convolutional layer proposed in this paper as a reference, the model performance of the 6-layer convolutional layer and 12-layer convolutional layer were compared. It could be found that when all three models were trained 30 times, the classification accuracy of each model on the test data could reach more than 97%. The difference was that with the increase of convolutional layers, the model converged faster, and the classification accuracy of training data was higher. It was indicated that the training accuracy with 12 convolutional layers was lower than that of 10 convolutional layers. Of course, a larger number of convolutional layers play an important role in the case of big data. Considering that the model would be applied to lightning sensors to classify lightning signals in real-time, due to the impact of embedded processor performance factors, we had to make a trade-off between the size of the model parameters and the processing speed. Based on the above considerations, a 10-layer convolutional neural network was adopted.

### 5.3. Real-Time Analysis

The method proposed in this paper would be applied to the three-dimensional lightning detection equipment deployed by the Institute of Electrical Engineering, Chinese Academy of Sciences in China, instead of the traditional waveform classification method. In order to evaluate the real-time performance of the 1D-CNN designed in this paper, we tested the model on a PC and an embedded platform, respectively. The model was executed on a 64-bit Windows 10 laptop (processor: Intel Core i7 CPU (@ 2.2 GHz)) using Tensorflow+Keras in Spyder open source cross-platform integrated development environment for scientific programming in Python. According to the test dataset, the time required for the model to execute the classification algorithm for 50,000 lightning waveforms was measured. The average time for the model to classify a waveform in the Spyder environment was 378.2 μs.

**Figure 11:**
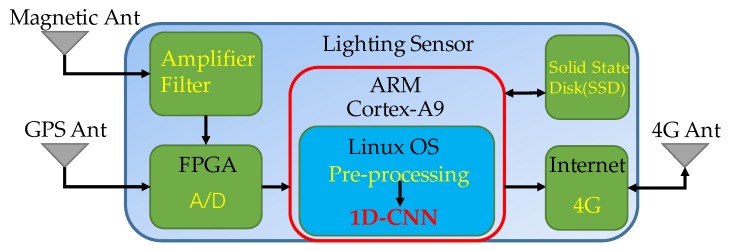
The system block diagram of the lightning sensor.

For embedded platforms, we are currently using a Cortex-A9 processor with a CPU operating frequency of 800 MHz. FPGA (Field-Programmable Gate Array) performs data acquisition and triggers the time labeling of lightning waveforms. The ARM processor performs tasks, such as data preprocessing, waveform classification, and calculating signal arrival time. After the data analysis is completed, it would be saved to the local solid-state hard disk, and the processing results would be transmitted to the server located at the Institute of Electrical Engineering, Chinese Academy of Sciences, via the network. The system block diagram of the lightning sensor is shown in Figure 11. We transplanted the 1D-CNN model that has been trained on the PC to the embedded processor. The average time for this embedded system to process a waveform was 4.12 ms. Considering the global average of 30 to 100 lightning occurrences per second, the calculation cost required by this model was significantly less than the frequency of lightning occurrences, indicating that the algorithm could be applied to real-time lightning signal analysis.

## 6. Conclusions

Based on a large amount of lightning waveform data, 10 types of 50,000 waveform samples were extracted to form a lightning dataset. A 1D-CNN lightning signal classification model was built using the deep learning method.

Compared with the simple classification method of cloud flash and cloud ground flash based on statistical methods, the 1D-CNN model could realize multiple classifications of lightning signals. Among them, a 1-D convolutional layer containing multiple filters (convolution kernels) extracts the characteristics of lightning signals at different time scales, multiple pooling layers down-sample the lightning signals, and summarize global information through a fully connected layer. The dataset comes from lightning signals collected at different sites, different times, and during different lightning processes. The diversity of samples makes the model have good generalization and adaptability. For 10 types of lightning datasets, the overall recognition accuracy of the model was as high as 99.11%. Compared with the classification method using statistical rules, the classification accuracy rate was only 55.66%, which was far lower than the lightning classification model using the deep neural network method. For the selected thunderstorm process, the overall model recognition accuracy was 97.55%, and for most categories, the recognition accuracy was above 97%.

Under the condition of having a large amount of lightning data, we also found more other types of lightning signals by using this model. In future research, we would try to fine-tune these signals to provide a more comprehensive and reliable classification of lightning signals. Since distinguishing between cloud flashes and cloud ground flashes has been an important indicator for lightning monitoring and research, and accurate lightning classification can significantly improve the positioning accuracy of the lightning positioning network, the application of deep learning in the classification of lightning signals proposed in this paper has a bright future.

## Figures and Tables

**Table 1:** Model parameters.

No.	Layer	Filter Number	Kernel Size	Pooling Window Size	Padding	Stride	Activation Function	Output Shape
1	Input	/	/	/	/	/	/	(1000,1)
2	1D-Conv	16	32	/	√	/	ReLU	(1000,16)
3	1D- Conv	16	32	/	√	/	ReLU	(1000,16)
4	Max-Pooling	/	/	2	×	2	/	(500,16)
5	1D- Conv	32	32	/	√	/	ReLU	(500,32)
6	1D- Conv	32	32	/	√	/	ReLU	(500,32)
7	Max-Pooling	/	/	2	×	2	/	(250,32)
8	1D- Conv	64	16	/	√	/	ReLU	(250,64)
9	1D- Conv	64	16	/	√	/	ReLU	(250,64)
10	Max-Pooling	/	/	2	×	2	/	(125,64)
11	1D- Conv	128	8	/	√	/	ReLU	(125,128)
12	1D- Conv	128	8	/	√	/	ReLU	(125,128)
13	Max-Pooling	/	/	5	×	5	/	(25,128)
14	1D- Conv	256	3	/	√	/	ReLU	(25,256)
15	1D- Conv	256	3	/	√	/	ReLU	(25,256)
16	Mean-Pooling	/	/	5	×	5	/	256
17	Dense	/	/	/	/	/	Softmax	10

* 1D-Conv: 1D convolutional layer. ReLU: Rectified Linear Unit.

**Table 2:** Five-fold cross-validation for different types of classification accuracy.

K	−CG (%)	CG-IR (%)	NBR (%)	+CG (%)	MP (%)	+PBP (%)	−PBP (%)	−NBE (%)	+NBE (%)	SW (%)	Ave (%)
1	98.84	99.40	98.88	99.64	99.58	100.00	96.50	99.80	98.16	99.82	99.06
2	96.16	99.10	94.98	99.86	99.86	99.94	98.20	99.80	99.30	99.00	98.62
3	99.04	99.34	99.36	99.96	99.82	99.94	98.36	99.92	99.18	99.84	99.47
4	97.74	99.32	98.64	99.58	99.84	99.96	98.42	99.92	99.36	99.80	99.26
5	98.52	98.82	98.48	99.16	99.74	99.92	97.68	99.88	99.04	99.88	99.11
Ave	98.06	99.20	98.07	99.64	99.77	99.95	97.83	99.86	99.01	99.67	99.11

* K: the *K*th cross-validation.

**Table 3:** The classification accuracy of the model on the test set.

	−CG	CG-IR	NBE	+CG	MP	+PBP	−PBP	−NBE	+NBE	SW	Total
TP	1321	1852	1180	179	260	417	830	729	298	2345	9411
NP	23	28	84	32	8	4	32	3	0	22	236
Acc(%)	98.29	98.51	93.35	84.83	97.01	99.05	96.29	99.59	100.00	99.07	97.55

**Table 4:** Results of statistical classification.

Type	CC	CG	+NBE/−NBE	Other	Acc (%)
−CG	2232	**2752**	0	16	55.04
CG-IR	4196	**582**	0	222	11.64
NBE	**4049**	1	0	950	80.98
+CG	0	**3436**	0	1564	68.72
MP	**522**	3	0	4475	10.44
+PBP	**1202**	0	2	3796	24.04
−PBP	**3507**	1	1	1491	70.14
−NBE	0	4	**3206**	1790	64.12
+NBE	0	904	**3953**	143	79.06
SW	**4620**	66	0	314	92.40
Average					55.66

* Bold numbers indicate the correct classification.

## Supplementary Materials

The test data and 1D-CNN lightning classification model are available online at https://github.com/jqwang1993/lightning-dataset.
